# Relationship between the state of tongue hygiene and the number of residual teeth in convalescent-ward inpatients, cross- sectional study

**DOI:** 10.1186/s12903-019-0869-3

**Published:** 2019-08-06

**Authors:** Kanako Hayashi, Maya Izumi, Ayaka Isobe, Yuhei Mastuda, Sumio Akifusa

**Affiliations:** 1 School of Oral Health Sciences, Kyushu Dental University, 2-6-1, Manazuru, Kokurakita-ku, Kitakyushu-shi, Fukuoka 803-8580 Japan; 2 Department of Oral and Maxillofacial Surgery, Faculty of Medicine, Shimane University, 89-1, En-yacho, Izumo-shi, Shimane 693-8501 Japan

**Keywords:** Convalescent ward, Inpatient, OHAT, Saliva, Tongue

## Abstract

**Background:**

The changed disease landscape in Japan because of an increasing aging population has contributed to an increase in convalescent inpatients, warranting important considerations of their oral care needs. However, information on the oral state of these inpatients is scarce. We evaluated the correlation between the number of residual teeth and tongue hygiene state in these inpatients.

**Methods:**

This cross-sectional study included convalescent-ward inpatients, aged 34–100 years. The study was conducted between April 2017 and March 2018 in Kitakyushu, Japan. Data regarding age, sex, number of residual teeth, odontotherapy requirement, medications with oral side effects, and the reason for hospitalization, were collected. Oral hygiene level was assessed using the Oral Health Assessment Tool (OHAT). The correlation between each element of OHAT and the number of residual teeth was analyzed using Pearson’s correlation analysis. The risk of a remarkable tongue state was analyzed using binominal logistic regression analysis.

**Results:**

Correlations were observed between the number of residual teeth and OHAT subscales, including tongue, saliva, and dentures. A significantly higher percentage of inpatients with ≤19 teeth had a tongue state score of 1 or higher, compared with those with ≥20 teeth. (78.6% vs 57.7%, *p* = 0.047). In inpatients with ≥20 teeth, the remarkable saliva state significantly increased the risk of the remarkable tongue state by 10.49-fold (95% confidence interval = 2.86–38.51), after adjusting for potential confounders.

**Conclusion:**

Poor tongue hygiene is associated with the number of teeth and salivary state in convalescent-ward inpatients. Inpatients with ≤19 teeth had a higher risk of poor tongue hygiene, regardless of the salivary condition, as assessed using OHAT.

## Background

With the changing disease landscape in Japan, the requirement for convalescence medical care continues to increase. Although the incidence rates are declining every year, the total number of patients suffering from stroke was 1,179,000 in 2016, and the estimated rate of inpatients/outpatients with stroke per 100,000 population was 199 [1], which is still a large numbers. Moreover, there is a high prevalence of locomotor disorders due to osteoporosis in the aging population [2]. Patients with stroke, locomotion disorders, and postoperative disuse atrophy are the main types of patients in the recovery phase rehabilitation ward, a ward for convalescence care. Unlike young adults and children, recovery after an acute illness or surgery is more prolonged in older adults. Hence, the need for convalescent hospital settings is very important for older patients. To prevent aspiration pneumonitis and facilitate earlier discharge, oral hygiene intervention is critical for inpatients in convalescent hospital settings. However, it has been observed that daily oral care interventions provided by nurses of the convalescent ward is not adequate and is not performed in an systematic manner [3], suggesting that oral hygiene may be poor in these inpatients.

In hospital wards, the oral assessment guide (OAG) is a well-known tool used to assess the state of oral hygiene [4]. Andersson modified one of the categories (saliva) of assessment in the OAG and thus designated it as the revised OAG (ROAG) [5]. Another tool, the Oral Hygiene Assessment Tool (OHAT) is also used widely [6]. While the OAG was developed to assess the oral state of patients on cancer chemotherapy, the OHAT was developed for older adults in nursing homes or receiving care at home; hence, the OHAT also includes a category for masticatory ability. Operational definitions of each category of OHAT with photographs are available, and scores can be easily determined even by non-dental professionals. A score of 1 or 2 for any of the categories mandates referral to an oral health professional [7].

Tooth loss has been known to influence food choice [8], diet [9], and nutrition intake [10]. The impact of tooth loss on weight is controversial; whereas tooth loss could lead to weight gain/obesity until the late middle age [11], it could cause weight loss in old age [12]. Tooth loss substantially impacts the quality of life and mental health [13, 14]. Tooth loss has been identified as a predictor of shortened longevity [15], and is associated with the risk of developing systemic diseases, such as cardiovascular disease [16], cerebrovascular disease [17], cancer [18], and cognitive impairment [19]. Considering that the causes of tooth loss were mainly dental caries and periodontitis, the number of remaining teeth could be a possible predictor of the state of oral hygiene. The purpose of this study was to investigate if there was a correlation between the number of residual teeth and the state of oral hygiene as assessed by the OHAT in inpatients of the convalescent ward.

## Methods

### Design and participants

The cross-sectional study was carried out in two hospitals providing convalescence medical care, between April 2017 and March 2018 in Kitakyushu, Fukuoka prefecture, Japan. A total of 94 inpatients (39 men and 55 women) participated in the study. Persons with less than 5 points in the communication score of functional independence measure (FIM) were excluded. The study was approved by the Medical Ethics Committee of Kyushu Dental University (No. 16–42). Written informed consent was obtained after the details of the study were explained to the participants.

### Data collection

Participant demographic and clinical data, including age, sex, primary cause of hospitalization, prescribed medications, FIM, and dental status (whether receiving odontotherapy), were collected from medical records. The persons whose cognitive function of FIM was under 5 points were excluded. The total number of teeth present was recorded by registered dental hygienists. The OHAT has been developed for use by non-dental professionals such as nurses, personal care attendants, allied health, and medical professionals [6]. The OHAT consists of eight categories (‘lips’, ‘tongue’, ‘gums and tissues’, ‘saliva’, ‘natural teeth’, ‘dentures’, ‘oral cleanliness’, and ‘dental pain’) with three possible scores (0: healthy, 1: some observed changes, and 2: unhealthy). To facilitate OHAT use, a Japanese manual with descriptions of the different scores for each category was used. Scoring using the criterion of ‘tongue state’ on original assessment was as follows: score 0 (healthy) - normal, moist, rough, and pink; score 1 (changes) - patchy, fissured, red, and coated; score 2 (unhealthy) - red and/or white patch that is ulcerated or swollen. Scoring using the criterion of ‘saliva’ was as follows: score 0 (healthy) - moist tissues, watery, and free flowing saliva; score 1 (changes) - dry, sticky tissues, little saliva present; score 2 (unhealthy) - tissues parched and red, very little/no saliva present, thick saliva [6]. Although, originally, scoring using the criterion of saliva includes self-reported dryness of mouth, this criterion was excluded because most inpatients, especially older patients, complain of dry mouth. Hence, the state of saliva was scored only by objective inspection.

### Statistical analysis

Values represent mean ± standard deviation (SD). For statistical analysis, we classified the score for each OHAT subscale as either 0 or ≥ 1, and the number of residual teeth was classified as ≤19 or ≥ 20. Age, sex, receiving odontotherapy, primary cause of hospitalization, receiving medications with oral side-effects, and difference in the participants’ hospital-of-admission were considered as potential confounders. Logistic regression analysis was used to analyze the factors correlated with the state of tongue hygiene. Statistical analysis was performed using SPSS (version 22; SPSS Japan Inc., Tokyo, Japan). We calculated two-tailed *p*-values in all analyses. An alpha level of 0.05 was considered significant.

## Results

A total of 94 inpatients (39 men and 55 women) were analyzed. The participants were aged between 34 and 100 years with a mean age of 70.2 ± 16.2 years. The mean number of teeth was 18.6 ± 10.3; there were 20.4 ± 10.9 teeth in men and 17.4 ± 9.7 teeth in women, indicating no significant difference with regard to sex (*p* <  0.165 using t test). Table 1 shows the correlation between the number of residual teeth and the OHAT subscale scores. Correlations were observed between the number of residual teeth and OHAT scores related to tongue (r = − 0.221, *p* <  0.05), saliva (r = − 0.210, p <  0.05), and dentures (r = − 0.305, *p* <  0.01). There was no correlation between the scores of the tongue and dentures (r = − 0.031, *p* < 0.77).

**Table 1:** Correlation between number of residual teeth and OHAT subscales analyzed with Pearson coefficient of correlation

	variables	1	2	3	4	5	6	7	8
1	tooth number	–							
2	lip	0.034	–						
3	tongue	−0.221^*^	−0.078	–					
4	gum/tissue	−0.049	0.173	0.048	–				
5	saliva	−0.210^*^	0.066	0.394^**^	0.196	–			
6	natural teeth	0.117	0.114	−.233^*^	0.489^**^	−0.123	–		
7	denture	−0.305^**^	0.143	0.031	0.046	0.238^*^	−0.033	–	
8	oral cleanliness	0.192	0.059	−0.044	0.497^**^	0.261^*^	0.432^**^	0.125	–
9	dental pain	−0.072	0.252^*^	0.160	0.355^**^	0.202	0.084	0.213*	0.180

*: *p* < 0.05, **: *p* < 0.01

Subsequently, we divided the participants into two cohorts based on the number of teeth: participants with ≤19 teeth (44.7%) and ≥ 20 teeth (55.3%). Each subscale of OHAT with a score ≥ 1 was defined as a remarkable case. Table 2 indicates that the ratio of remarkable tongue state in participants with ≤19 teeth was significantly higher than that in those with ≥20 teeth (78.6% vs 57.7%, *p* = 0.047). With regard to the mean total scores of OHAT, there was no significant difference between the cohorts with ≤19 teeth (3.6 ± 1.9) and ≥ 20 teeth (2.9 ± 2.2, *p* = 0.134).

**Table 2:** Ratio of each OHAT subscale divided by number of residual teeth in inpatients

OHAT subscales		Residual tooth number				*p* value
		< 19 N (%)		> 20 N (%)		
lip	0	41	(97.6)	49	(94.2)	0.626
	> 1	1	(2.4)	3	(5.8)	
tongue	0	9	(21.4)	22	(42.3)	0.047
	> 1	33	(78.6)	30	(57.7)	
gum/tissue	0	20	(47.6)	31	(59.6)	0.300
	> 1	22	(52.4)	21	(40.4)	
saliva	0	13	(31.0)	25	(48.1)	0.139
	> 1	29	(69.0)	27	(51.9)	
natural teeth	0	31	(73.8)	33	(63.5)	0.374
	> 1	11	(26.2)	19	(36.5)	
denture	0	27	(64.3)	48	(92.3)	0.001
	> 1	15	(35.7)	4	(7.7)	
oral cleanliness	0	20	(47.6)	21	(40.4)	0.534
	> 1	22	(52.4)	31	(59.6)	
dental pain	0	39	(92.9)	49	(94.2)	1.000
	> 1	3	(7.1)	3	(5.8)	
mean score of OHAT		3.6 ± 1.9		2.9 ± 2.2		0.134

To evaluate the effect of the state of saliva on the state of tongue hygiene, we divided the participants into two cohorts by the state of saliva (Table 3). Ratio of remarkable tongue state in participants with remarkable saliva state was significantly higher than those with normal saliva (42.1% vs 83.9%, *p* < 0.001). These correlations were also observed in participants with ≥20 teeth (32.0% vs 81.5%, p < 0.001) but not in those with ≤19 teeth (61.5% vs 86.2% *p* = 0.084).

**Table 3:** Difference of tongue and saliva states by number of residual teeth

tooth number	tongue	saliva				*p* value
		0 N (%)		> 1 N (%)		
total	0	22	(57.9)	9	(16.1)	> 0.001
	> 1	16	(42.1)	47	(83.9)	
< 19	0	5	(38.5)	4	(13.8)	0.084
	> 1	8	(61.5)	25	(86.2)	
> 20	0	17	(68.0)	5	(18.5)	> 0.001
	> 1	8	(32.0)	22	(81.5)	

In this study, data were collected from two hospitals. There was heterogeneity in certain characteristics between the two hospitals. In one hospital, the ratio of primary disease at admission was 82.5% orthopedic disease and 17.1% stroke. In the other hospital, the ratio was 33.9% orthopedic disease, 59.3% stroke, and 6.8% other diseases (*p* < 0.001). The OHAT score of the participants from one ward (4 [1–12]) was significantly higher than that of the other ward (3 [0–9], *p* = 0.009).

The results of logistic regression analysis for the association between the state of tongue hygiene and the state of saliva are shown in Table 4. In the univariate model, participants with remarkable saliva had significantly higher (7.18 times) risk of deterioration of the state of tongue hygiene compared to the participants with normal state of saliva (*p* < 0.001). In the multivariate model, the state of saliva was associated with higher odds of deterioration of tongue hygiene after adjustment for covariates including age, sex, receiving odontotherapy, primary cause of hospitalization, receiving medications with oral side-effects, and difference in the participants’ hospital-of-admission (odds ratio = 10.49, 95% confidence interval: 2.86–38.51, p < 0.001). When divided by the number of residual teeth, these associations between tongue and saliva were also observed in the cohort having ≥20 teeth (adjusted odds ratio = 22.27, confidence interval: 2.42–205.28, *p* = 0.006) but not in the cohort having ≤19 teeth.

**Table 4:** Association between states of tongue and saliva divided by number of residual teeth determined using logistic regression

tooth number	saliva	Crude OR (95% CI)	*p* value	Adjusted ^a^ OR (95% CI)	*p* value
total	0	1.00 (ref.)	< 0.001	1.00 (ref.)	< 0.001
	> 1	7.18 (2.75–18.77)		10.49 (2.86–38.51)	
< 19	0	1.00 (ref.)	0.092	1.00 (ref.)	0.068
	> 1	3.75 (0.81–17.48)		6.26 (0.87–44.91)	
> 20	0	1.00 (ref.)	0.002	1.00 (ref.)	0.006
	> 1	7.70 (2.09–28.34)		22.27 (2.42–205.28)	

*OR* odds ratio, *CI* confidence interval

^a^adjusted with age, sex, receiving odontotherapy, primary cause for hospitalization, receiving medicines with oral side-effect, hospital

## Discussion

The results of the present study suggest that the need for intervention in relation to tongue hygiene differed based on the number of residual teeth in convalescent-ward patients. Inpatients with ≤19 teeth required tongue-hygiene care regardless of the condition of the saliva. On the other hand, in patients with ≥20 teeth, intervention was required only if the salivary condition was remarkable. To the best of our knowledge, the present study is the first to report the relationship between the status of tongue hygiene and the number of residual teeth in inpatients.

The reason for the relationship between the state of tongue hygiene and the number of residual teeth is currently not obvious. Many studies have demonstrated that tongue coating due to inadequate tongue hygiene can cause halitosis [20–22]. The dorsum of the tongue is the main site of origin of volatile sulfur compounds in patients with periodontitis [23]. A previous study describing the comprehensive microflora profile of tongue coating determined using terminal restriction fragment length polymorphism analysis indicated a close relationship between tongue coating and pneumonia [24]. Wiping the oral mucosa, including the tongue dorsum, with a chlorhexidine-soaked sponge brush decreased the number of opportunistic pathogens in the pharyngeal region [25]. These findings suggest that reducing the amount of coating on the tongue can possibly decrease the risk of aspiration pneumonia. A previous study reported that the presence of tongue coating is related to several factors like smoking, wearing dentures, periodontal status, and dietary habits, however, it did not include the condition of the saliva [22]. In this study, the state of tongue hygiene had no correlation with wearing dentures. It is possible that denture care may have been well managed by the nurses and/or healthcare professionals in the hospitals in this study.

The relationship between saliva and the number of residual teeth in older adults is controversial. One previous report demonstrated that there was no significant correlation between the number of residual teeth and the stimulated salivary flow rate (r = 0.028, *p* = 0.316) in Japanese individuals over the age of 60 years [26], which is consistent with our results. In contrast, another study reported that there is an inverse correlation between the number of missing teeth and the stimulated salivary flow and salivary buffer capacity in Mexicans over the age of 60 years [27], suggesting that salivary malfunctions could cause tooth loss. Although the reasons for this discrepancy are not obvious, the mean salivary flow rate in the former study was 0.97 mL/min, and in the latter was 0.75 ml/min, suggesting differences in the participants.

To preserve a healthy mucosal and tongue surface, adequate salivary flow and optimum salivary composition are required. An earlier study demonstrated that the pH of saliva tends to be acidic while the pH of tongue coating tends to be alkaline [22]. This suggests that there is a quantitative and qualitative association between the tongue coating and saliva [22]. Secretory-immunoglobulin A levels in the saliva are known to have a correlation with the amount of tongue coating [28]. Saliva viscosity and tongue coating are associated with oral malodor [29]. Low flow rate of stimulated saliva is strongly associated with the risk of developing an atrophic tongue [30]. Taking all of this into consideration, inpatients with a deteriorated salivary state need appropriate intervention measures to maintain oral hygiene and a healthy tongue surface.

The presence of ≥20 remaining teeth has a positive impact on health status, especially in older adults [31]. The number of remaining teeth continues to increase in the 70 years and above age group in Japan, and the mean residual number of teeth in older adults was 14.0 and the percentage of the 70 years and above age group with ≥20 teeth was 52.3% in 2016 [32]. Tooth loss is now shown to affect longevity and life expectancy [15]. Octogenarians with ≥20 teeth had higher daily activity and life satisfaction [33]. A large scale survey in Japan demonstrated that individuals with ≥20 teeth had lower mortality risk (0.58 times) and morbidity risk of diabetes (0.52 times) than did edentulous individuals [33].

The reason for the association between lower number of teeth and tongue hygiene levels in inpatients of convalescent wards may be that as oral health habits are closely related with number of teeth [34], inpatients with < 19 teeth had worse habits, which may have had an impact on tongue hygiene levels.

### Limitations

This study has several limitations. First, not all the potential confounding factors, such as smoking history, education level, and socioeconomic status, were considered. One previous study indicated that smoking increases the salivary flow rate [35]. Second, we did not evaluate oral diseases such as dental caries and periodontitis. A relationship between tongue coating and periodontal lesions due to associated microbial flora has been identified [20]. Third, the participants of this study were inpatients of the convalescent wards of two hospitals. The major causes of hospitalization in these wards were orthopedic disorders and cerebrovascular diseases. The effect of other illnesses such as disuse atrophy or cardiac infarction on tongue hygiene conditions is unclear. Fourth, the intra-examiner reliability of OHAT cannot be calculated because data collection was performed only once. Although the dental hygienist was well trained for the procedure, validation of the intra-examiner reliability may be required in order to increase the reliability of OHAT examination.

## Conclusion

This is the first report showing a positive correlation between the state of tongue hygiene and the number of residual teeth in convalescent-ward inpatients. In inpatients with ≥20 teeth, deterioration of the salivary state critically influenced the state of tongue hygiene. In contrast, inpatients with ≤19 teeth had a higher risk of poor tongue hygiene regardless of the state of saliva. These findings suggest that the number of residual teeth could be used as a parameter to determine intervention plans in daily oral care by non-dental professionals in convalescent wards.

## Data Availability

The datasets used and/or analyzed during the current study are available from the corresponding author on reasonable request.

## Abbreviations

FIM: functional independence measure
OHAT: Oral Hygiene Assessment Tool
